# The Molecular Pathways of Pyroptosis in Atherosclerosis

**DOI:** 10.3389/fcell.2022.824165

**Published:** 2022-02-14

**Authors:** Dan Song, Manman Li, Xue Yu, Yuqin Wang, Jiaying Fan, Wei Yang, Liming Yang, Hong Li

**Affiliations:** ^1^ Department of Pathophysiology, School of Basic Medical Sciences, Harbin Medical University, Harbin, China; ^2^ Department of Pathophysiology, Harbin Medical University-Daqing, Daqing, China

**Keywords:** pyroptosis, atherosclerosis, SIRT, miRNA, NF-κB, AMPK, MAPK

## Abstract

Atherosclerosis (AS) is a chronic inflammatory disease seriously endangering human health, whose occurrence and development is related to many factors. Pyroptosis is a recently identified novel programmed cell death associated with an inflammatory response and involved in the formation and progression of AS by activating different signaling pathways. Protein modifications of the sirtuin family and microRNAs (miRNAs) can directly or indirectly affect pyroptosis-related molecules. It is important to link atherosclerosis, thermogenesis and molecular modifications. This article will systematically review the molecular pathways of pyroptosis in AS, which can provide a new perspective for AS prevention and treatment.

## Introduction

AS, a chronic inflammatory lesion (Ray et al., 2019), usually remains stable for several years, but can rapidly become unstable, rupture and trigger thrombosis. Thus, in addition to limiting the lumen, the presence of atherosclerotic plaque is associated with an increased risk of acute cardiovascular events such as myocardial infarction (MI) and stroke (Emini Veseli et al., 2017). It is also a major reason for the development of vascular disease and a leading contributor to death around the world, with about 7.2 million deaths each year, and the prevalence is expected to increase about 18% by 2030 (Valanti et al., 2021). Many studies have shown that the pyroptosis that accompanies the inflammatory response can be involved in the whole process of AS development (Xu et al., 2018; Qun Wang et al., 2020). Stimuli that promote the development of AS, such as nicotine, hyperlipidemia, oxidized modified low-density lipoprotein (ox-LDL), and cholesterol crystals (CC-) can induce the pyroptosis-associated inflammasome and caspase-1 through different pathways (Zhaolin et al., 2019a). Endothelial cells, which act as a barrier between the blood and the vessel wall interface, are the first cells in the circulatory system to be exposed to danger signals, and their impaired function is often considered to be the initial stage of AS (Gimbrone and García-Cardeña, 2016; Paone et al., 2019). It has been shown that nicotine in tobacco increases reactive oxygen species (ROS) production and activates the NLRP3 inflammasome, which promotes caspase-1 maturation and the production and release of IL-1β and IL-18, triggering inflammation and endothelial cells pyroptosis and promoting the AS process (Arend et al., 2008; Wu et al., 2018).

## Atherosclerosis

AS is an inflammatory process that mainly includes endothelial damage, lipid deposition, macrophage activation, phagocytosis, inflammatory response, foam cell formation, oxidative modifications, and smooth muscle cell migration (Ross, 1999). During the development of AS, death of vascular cells such as endothelial cells (ECs), macrophages, and smooth muscle cells (SMCs) is common (Xu et al., 2018). AS begins with vascular endothelial injury and subsequently progresses to chronic inflammation and atherosclerotic plaque formation (Zhou et al., 2021). The early stage of atherosclerotic lesion development is called “fatty streak” (Poznyak et al., 2020), and in the initial stage of atherogenesis, ox-LDL causes endothelial cell damage and dysfunction, which can lead to a variety of human health-threatening diseases such as stroke, and further promotes monocyte binding to sites of endothelial wall damage (Moreno et al., 1994), in the endothelium they differentiate into macrophages and internalize modified lipoproteins to become foam cells (Libby et al., 2019), and activated endothelial cells and macrophages secrete a variety of chemokines and growth factors that promote inflammation and ROS production (Kattoor et al., 2017; Libby et al., 2019). They then act on adjacent smooth muscle cells, inducing their proliferation and synthesis of extracellular matrix components within the endothelial lumen, resulting in fibromuscular plaques (Garcia and Blesso, 2021). The plaque is composed of a lipid core, foam cells and collagen fibrous cap. In the advanced stages of AS, rupture of vulnerable plaques exposes their thrombogenic compounds, which leads to thrombosis (Kalz et al., 2014). Advanced atherosclerotic lesions are characterized by large necrotic cores with thin fibrous caps, cholesterol deposition, inflammatory cells and calcification (Yahagi et al., 2016). Macrophage-derived proteases, which can destabilize plaques. In addition, during plaque progression, small micro-vessels begin to form in the plaque during hypoxia to provide nutrition to the plaque. In addition, during plaque progression, small micro-vessels begin to form in the plaque during hypoxia, and these micro-vessels remain immature in the atherosclerotic environment, which may lead to intraplaque hemorrhage and damaged plaque rupture (Foks and Bot, 2017).

## Pyroptosis

Pyroptosis is a newly discovered pattern of programmed cellular necrosis (Bin He et al., 2021), characterized by cell swelling, plasma membrane rupture, and massive production and release of pro-inflammatory factors, triggering a cascade of amplified inflammatory responses, often induced by endogenous injury or bacterial and viral infections (Zhaolin et al., 2019a). The term pyroptosis was first introduced in 2001 by Cookson et al. who found that the rapid form of macrophage death caused by Salmonella was closely resembling necrosis, but differed in that this mode of death was mainly dependent on the caspase-1 (Cookson and Brennan, 2001). Later, as related studies continued, the explanation for pyroptosis was gradually completed.

Unlike other programmed cell death, pyroptosis has specific morphological features and a unique activation mechanism of the pyroptotic pathway (Zhaolin et al., 2019a). Pyroptosis is an important natural immune response of the body and plays an important role in the fight against infection. The basic process is that when multiple pathological factors such as oxidative stress, hyperglycemia and inflammation stimulate the organism, cells receive endogenous and exogenous danger signals to induce intracellular formation of inflammatory vesicles, activating caspase-1, that in return mediates the maturation and extracellular production of pro-inflammatory factors via gasdermin D (GSDMD) (Vande Walle and Lamkanfi, 2016; Barnett and Ting, 2020). During this period, necrosis-like cell membrane pore formation, cell swelling and membrane rupture resulted in massive leakage of cytoplasmic contents as well as apoptotic-like nuclear condensation and DNA ladder breaks, while the integrity of the nucleus and mitochondria was maintained (Man et al., 2017; Broz et al., 2020).

**FIGURE 1:**
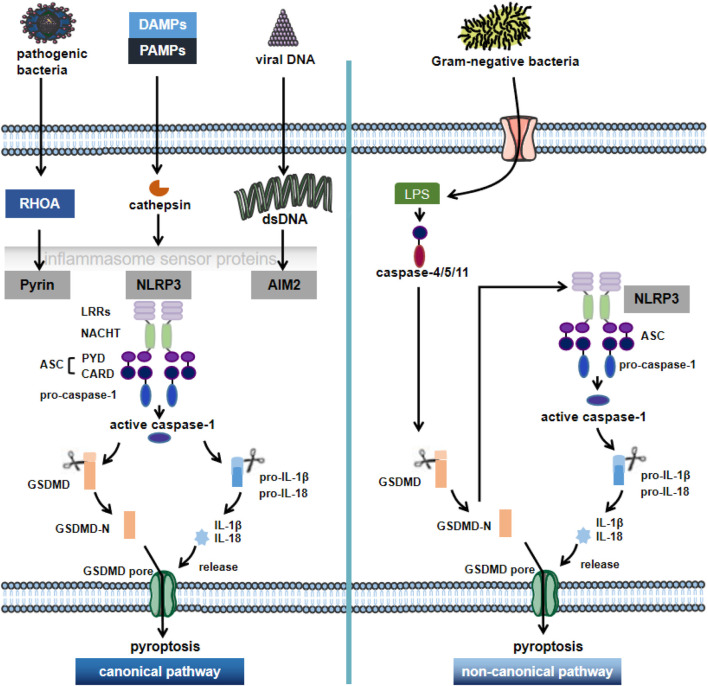
The canonical pathway and non-canonical pathway of pyroptosis. Pyroptosis is a novel pro-inflammatory regulator of cell death. Depending on the key molecular patterns involved in the activation and execution steps, they can be divided into the canonical pathway and non-canonical pathway. In the caspase1-dependent pyroptosis pathway, cells can activate inflammatory vesicles to trigger pyroptosis in response to multiple factors, activating the respective inflammatory vesicles (including NLRP3, AIM2 or pyrin) through the action of PAMPs and DAMPs; NLRP3 recruits ASC and pro-caspase-1 through various pathways, causing activation of caspase-1 and maturation and secretion of pro-inflammatory cytokines such as IL-1β and IL-18. GSDMD-N is formed by the cleavage of inflammatory cystathione, triggering the rupture of cell membranes and promoting the release of inflammatory factors, cell swelling and pytoptosis. In the non-canonical pathway, the Gram-negative bacterial cell wall fraction LPS activates caspase 4/5/11, which mediates pyroptosis by directly triggering pyroptosis through the cleavage of GSDMD. NLRP3: NOD-like receptor protein 3; AIM2: in melanoma 2; PAMPs: pathogen-associated molecular patterns; DAMPs: danger-associated molecular patterns; GSDMD: gasdermin D; LPS: lipopolysaccharide.

The molecular mechanisms of pyroptosis mainly include caspase-1-dependent canonical pathway and caspase-4/5/11-dependent non-canonical pathway (Liu and Sun, 2019) (Figure 1). The upstream of caspase-1 is inflammatory vesicles (Lee et al., 2016): including NLRP3, absence in melanoma 2 (AIM2), Pyrin structural domain (pyrin and HIN domain), etc. The most widely studied NLRP3 inflammatory vesicle, which plays an important role in the innate immune system and TLR4 induces inflammatory responses by nuclear factor kappa-light chain-enhancer of activated B (NF-κβ) and increasing the production of pro-inflammatory cytokines (Yu et al., 2019). Nowadays, binds to apoptosis-associated spot-like protein (ASC) sites through homotypic interactions after being activated in the presence of pathogen-associated molecular patterns (PAMPs) and danger-associated molecular patterns (DAMPs), among others, with the N-terminal PYD structural domain (Xu et al., 2018). ASC then recruits polymerized pro-caspase-1 through its CARD structural domain (caspase activation and recruitment domain) and induces its own cleavage to form caspase-1 maturation (p10/p20 tetramer). Activated caspase-1 shears inactive IL-1β precursors and IL-18 precursors, converting them into mature inflammatory factors, leading to pyroptosis (Qian et al., 2021). On the other hand, it cleaves GSDMD and oligomerizes the GSDMD-N-terminal fragment, which mediates the formation of membrane pores, resulting in cell swelling and lysis to further promote the release of inflammatory factors and intensify the inflammatory response, inducing pyroptosis (Winkler and Rösen-Wolff, 2015; Liu and Lieberman, 2017; Zahid et al., 2019).

In the non-canonical pathway of pyroptosis, lipopolysaccharide (LPS) is recognized by caspase-11 in mouse cells and by caspase-4 and caspase-5 in human cells; caspase-4/5/11, which is subsequently activated by receiving danger signals, can directly cleave GSDMD to trigger pyroptosis (Van Opdenbosch and Lamkanfi, 2019). Meanwhile, the GSDMD-N-terminal fragment activates NLRP3 inflammatory vesicles, which in turn activate caspase-1 and mediate the production of IL-1β and IL-18 to induce pyroptosis (Kun Wang et al., 2020).

The pyroptosis pathway is mainly regulated by inflammatory vesicles and caspase-1, triggering an inflammatory response (McKenzie et al., 2018). This process can occur anywhere in the body, with different sites leading to different outcomes. In the intrinsic immune cells, NOD-like receptors bind PAMPs or DAMPs ligands that are difficult to be cleared by cellular autophagy (Xiao He et al., 2021), activating the relevant inflammatory bodies and thus contributing to pyroptosis followed by a local or systemic inflammatory response to recruit more intrinsic immune cells to clear the excess PAMPs/DAMPs ligands (Qian et al., 2021). Therefore, as a form of inflammatory cell death, moderate pyroptosis can accelerate the immune response helping to resist pathogenic infections, maintain cellular homeostasis and exert a positive aspect of protecting the organism (Qun Wang et al., 2020). Of course, there is also a negative side to cell death. The high level of pyroptosis in some cells may lead to excessive activation of caspase-1 by abnormal inflammatory vesicles, resulting in a massive inflammatory response and the development of diseases such as AS (Winkler and Rösen-Wolff, 2015).

## Atherosclerosis and Pyroptosis

Trimethylamine N-oxide (TMAO), produced by phosphatidylcholine metabolism in the intestinal flora, is one of the most important factors threatening human vascular disease (Din et al., 2019). Recent studies have found that this product also upregulates the expression of caspase-1 and NLRP3, molecules associated with vascular endothelial cells pyroptosis, by inducing ROS production, further promoting the development of AS lesions in ApoE^−/-^ mice on a high-fat diet (Wu et al., 2020). Liu et al. found that TNF-α enhanced the expression of inter-cellular adhesion molecule-1 (ICAM-1), vascular cell adhesion molecule-1 (VCAM-1), and caspase-1, which in turn attracted monocytes to the intima and induced inflammatory responses and endothelial cells pyroptosis, which had important effects on the course of AS (Liu and Tie, 2019).

Altered endothelial permeability and functional impairment cause lipoprotein invasion modification to lipid deposition. As the disease progresses, macrophages that are unable to remove ox-LDL and CC- by autophagy undergo cell death and release large amounts of inflammatory factors, promoting the development of AS. Several reports confirm that ox-LDL-induced cell death is closely associated with pyroptosis functional molecules (Duewell et al., 2010; Peng et al., 2015). Duewell et al. showed that CC- and ox-LDL activate caspase-1 and NLRP3 inflammatory vesicles, leading to the release of IL-18 and IL-1β from macrophages, inducing inflammation and pyroptosis, and increasing the extent of plaque lesions (Peng et al., 2015). In addition, siRNA of NLRP3 expression eliminated the activation of IL-1β by ox-LDL and attenuated the development of AS in ApoE^−/-^ mice (Peng et al., 2015; Qiu et al., 2017). Ox-LDL is a risk factor involved in both AS and pyroptosis, exhibiting to some extent the correlation between AS and pyroptosis (Peluso et al., 2012; Zhaolin et al., 2019b).

Cell death in the vessel wall in the advanced stages of the disease causes disruption of membrane integrity and a continuous accumulation of lipids into a necrotic core, causing continued progression of the AS plaque (Libby et al., 2019). Pyroptosis, as a programmed cellular form, is also a cause of AS plaque instability (Qian et al., 2021). Pan et al. found an increase in plaque lesion area in high-fat fed ApoE^−/-^ mice with overexpression of inflammatory vesicles AIM2; and confirmed by *in vitro* experiments that the mechanism is that AIM2 mediates GSDMD through the caspase-1 pathway, increases vascular smooth muscle cell focalization, and affects AS plaques (Pan et al., 2018). Ox-LDL is a risk factor involved in both AS and pyroptosis, exhibiting to some extent the correlation between AS and pyroptosis (Kattoor et al., 2019). Relying on inflammatory vesicles and the caspase-1 pyroptosis pathway can perceive risk factors associated with AS and cause cells to pyroptosis during the pathological phase of AS, releasing inflammatory mediators that exacerbate the inflammatory response and contribute to the development of plaque instability (McKenzie et al., 2018; Van Opdenbosch and Lamkanfi, 2019). In summary, it can be seen that pyroptosis occupies an important position in all stages of AS development, and important functional molecules associated with pyroptosis can be used as markers to provide new approaches for the prevention and treatment of AS.

## The Molecular Pathways of Pyroptosis in Atherosclerosis

AS is a chronic disease, and pyroptosis is closely related to the development of AS through various signaling pathways such as nuclear factor kappa-light chain-enhancer NF-κβ, AMPK, and MAPK, etc. SIRT family, and miRNA by promoting the release of inflammatory factors, which may provide new therapeutic targets for the treatment of AS.

### Signaling Pathways

#### Nuclear Factor Kappa-Light Chain-Enhancer of Activated B

Among the various molecules and signaling pathways affected by NLRP3, NF-κβ cells is a nuclear factor (Dolcet et al., 2005). NF-κβ exists as a dimer and is to be engaged in the evolution and course of a variety of diseases related to pyroptosis and apoptosis (Emini Veseli et al., 2017). NF-κβ can be activated by a variety of factors, such as ROS and toll-like receptors (TLRs) (Mitchell et al., 2016; Yu-Ming Wang et al., 2019). ROS has been shown to have an important modulatory function in AS (Fang et al., 2021). Smoking is an important risk factor for AS, and Wu et al. showed that nicotine caused AS induction through ROS/NLRP3-mediated thermal apoptosis of endothelial cells (Wu et al., 2018; Xu et al., 2021). Activation of endothelial dysfunctional NLRP3 inflammatory vesicles requires activation of the TLR4/NF-κβ signaling pathway; subsequent upregulation of inflammatory vesicle components, including inactive NLRP3, pro-IL-1β, and pro-IL-18; followed by assembly of ASC, NLRP3, and pro-caspase-1 into a multiprotein complex (Leng et al., 2019; Yu et al., 2019). In the presence of obesity or dyslipidemia, an increase in serum free fatty acids provokes inflammation by a pathway that activates TLRs (Sokolova et al., 2017). TLR induces the development of AS by activating the transcription factor NF-κβ, which induces pyroptosis, thereby upregulating the transcription of the nod-like receptor family NLRP3 (Bauernfeind et al., 2009).

Studies have shown that NF-κβ is an essential transcription factor for GSDMD (Liu et al., 2017; Lei et al., 2018). The GSDMD is a critical effector of pyroptosis, and the C-terminal of the GSDMD is shown to automatically suppress the N-terminal pore-forming activity under normal cellular conditions (Shi et al., 2015). During activation of inflammatory vesicles by extracellular signals associated with focal death (like NLRP3 inflammatory vesicles), they then split and activate caspase-1, 4, -5 and -11 (Elliott and Sutterwala, 2015). Thus, activated caspase-1 cleaves and separates the N- and C-termini of GSDMD. It has been shown that the N-terminal fragment of GSDMD forms nanoscale pores in the cell membrane, leading to the release of pro-inflammatory substances and cell swelling (Man and Kanneganti, 2015; Lei et al., 2018). The NF-κβ-GSDMD axis is important in AS as a link between oxidative stress and pyroptosis.

#### AMP-Activated Protein Kinase

AMP-activated protein kinase (AMPK) a cellular energy sensor, is one of the key regulatory enzymes of cellular glycolipid metabolism (Yong et al., 2021), with anti-inflammatory and antioxidant activities (Hu et al., 2020). As an important regulator button of lipid metabolism, AMPK suppresses the production of malonyl-CoA by phosphorylating acetyl-CoA carboxylase (ACC), which allows active fatty acids to enter the mitochondria for oxidation or reduces the level of lipids (Fullerton et al., 2013). AMPK activation by suppressing inflammation by inhibiting pro-inflammatory signaling pathways and restricting the formation of specialized lipid products that elicit immune responses (Chandrasekar et al., 2008). AMPK is expressed in vascular endothelial cells and regulates vascular function (Ewart and Kennedy, 2011). CC- caused endothelial cell inflammation and pyroptosis play an integral role in the development of vascular diseases, especially atherosclerosis (Fang et al., 2021). Studies have shown that CC- is a key indicator of atherosclerotic plaque instability. CC- activates NLRP3 inflammatory vesicles, leading to IL-1β release and inducing rupture of necrotic cores and plaques, further promoting atherosclerotic lesions (Janoudi et al., 2016). NLRP3 inflammatory vesicles are large multiprotein complexes that regulate IL-1β production and are essential in the development of atherosclerosis. Activation of AMPK promotes the production of SIRT1 activator NAD+and increases the level of SIRT1(Zheng et al., 2020). Activates phosphorylated AMPK, reduces pro-inflammatory cytokines IL-6 and IL-1β, and decreases activation of NLRP3 inflammatory vesicles (Zhang et al., 2020). Thus, the AMPK pathway inhibits endothelial cell pyroptosis, protects plaque stability, and reduces adverse vascular lesions. Thus, the AMPK pathway inhibits endothelial cells pyroptosis, thereby stabilizing atherosclerotic plaques and reducing adverse cardiovascular events. The study of lipopolysaccharide-activated mouse macrophages revealed that ATP administration to mice activated the AMPK pathway in macrophages, accompanied by inflammation and pyroptosis, as indicated by cell membrane rupture and increased release of IL-1β and caspase-1 (Zha et al., 2016). Therefore, the study of AMPK has become an important target for the treatment of AS.

#### MAPK

p38 MAPK belongs to the mitogen-activated serine/threonine kinase family (Jiang et al., 2019). MAPK is activated in response to a variety of stimuli that include PAMPs and DAMPs that recruit pattern recognition receptors (PRRs), as well as factors associated with environmental stress (Kyriakis and Avruch, 2012; Reustle and Torzewski, 2018). Once activated, the MAPK pathway has significant effects on cellular physiology, and these stims are abundantly present in AS (Reustle and Torzewski, 2018). Natural LDL or its modification products may be inducers of p38 MAPK signaling in the early stages of AS (Zhu et al., 2001). Meanwhile, some studies found that LDL induced p38 MAPK phosphorylation and its nuclear translocation in endothelial cells and smooth muscle cells, promoting vascular calcification and focal death, further enhancing the abnormal proliferation of AS endothelium (Reustle and Torzewski, 2018). These summarized studies suggest that the p38 MAPK signaling pathway is involved in all phases of AS by activating different cellular responses. Furthermore normally, the NLRP3 inflammatory vesicle assembly trigger itself does not induce the so-called initiation step, which is the transcriptional induction of IL-1β and NLRP3 receptors, which can be induced by stimulation of TLRs (Arend et al., 2008). Luo et al. investigated a new brassinosteroid derivative 5-deoxy-rutaecarpine (R3) for the treatment of atherosclerosis and its molecular mechanism. R3 processing inhibits NLRP3 inflammatory vesicle activation in ApoE^−/-^ mice and ox-LDL-stimulated mouse macrophages by inhibiting NF-κβ and MAPK pathways (Luo et al., 2020). Ox-LDL-induced endothelial-mesenchymal transition (EndMT), endothelial cell inflammation and pyroptosis. HUVECs exposed to ox-LDL exhibit increased phosphorylation of ROS and p38 MAPK (Gong et al., 2019), leading to activation of MAPK and NF-κβ pathways and release of IL-1β(Wei Wang et al., 2019). The mechanism of MAPK inhibition of AS through pyroptosis still needs further study.

### Sirtuins

SIRTs (sirtuins) belong to the family of histone deacetylases (HDACs) (Chandrasekar et al., 2008). Their deacetylase activity is dependent on the key redox signaling molecule NAD^+^ (Ewart and Kennedy, 2011). In addition to deacetylases, some sirtuins have properties of adenosine diphosphate (ADP)-nucleotidase, demethylase, desuccinate lyase, or glutaminase (Winnik et al., 2015). It contains 7 enzyme activities (SIRT1-SIRT7) in mammals and represses gene transcription through epigenetic mechanisms (Mendes et al., 2017). The sirtuin family is highly expressed on blood vessels, promotes vascular homeostasis, and plays an important role in cardiovascular disease (CVD) (D'Onofrio et al., 2015). Of the seven SIRT subtypes, SIRT1 and SIRT3 had the most extensive cardiovascular manifestations (Sosnowska et al., 2017). The sirtuin family has been shown to influence the onset and progression of AS through the pyroptosis pathway.

#### Sirtuins1

SIRT1 is a member of the sirtuin family that regulates cellular functions such as cell survival, differentiation, metabolism, DNA repair, inflammation, neuroprotection, and can transfer acetyl groups from ε-n-acetyllysine on DNA histones to histones for transcriptional control (Jayachandran and Qu, 2021). Endogenous SIRT1 plays a key role in mediating cell death/survival (Matsushima and Sadoshima, 2015). Activation of SIRT1 can deacetylate acetyl groups on protein lysine residues, thereby regulating their biological functions (Chou et al., 2019). Inflammatory vesicles are involved in caspase-1 activation and maturation of IL-1β and IL-18, which are mainly released through pyroptosis (De Miguel et al., 2021). The most widely known inflammatory vesicle is NLRP3. Multiple studies have shown that NLRP3 inflammatory vesicles, IL-1β, and IL-18 play a decisive and important role in AS through pyroptosis (McKenzie et al., 2018). Arioz, B.I et al. found that NLRP3 inflammatory vesicles stabilize atherosclerotic plaques by activating nuclear factor red lineage 2-related factor 2 (Nrf2) and SIRT1(Arioz et al., 2019).

Activation of SIRT1 inhibits NLRP3 activation and subsequent caspase-1 cleavage and IL-1β secretion (Li et al., 2017). ECs damage forms the early stages of AS and contributes to atherosclerotic plaque formation, progression, and complications (Libby et al., 2019). In recent years, the activation of NLRP3 inflammatory vesicles in vascular endothelial cells is the beginning of the inflammatory response in endothelial cells (Li et al., 2017; Jia et al., 2019). Here, it was demonstrated that SIRT1 inhibited the activation of NLRP3 inflammatory vesicles, which can treat AS by reducing the expression of inflammatory vesicles and inhibiting the maturation of caspase-1 and IL-1β(Li et al., 2017). VSMCs are located in the middle layer of arteries and play a key role in maintaining the normal physiological function of blood vessels (Arioz et al., 2019). Abnormal VSMCs are associated with vascular diseases (VDs), VSMCs are involved in almost all progress of AS (Zhang et al., 2016). Meanwhile, macrophages play an important role in all stages of AS (Groh et al., 2018). From the onset of AS to its rupture and then to the regression and disappearance of the lesion (Tabas and Bornfeldt, 2016; Groh et al., 2018). Macrophages within plaques are mainly derived from monocytes in the blood, but it has also been shown that macrophages within plaques can also be derived from smooth muscle cells (Tabas and Bornfeldt, 2016). Oh et al. found that Pyrogallol-Phloroglucinol-6,6-Bieckol (PPB) significantly hindered monocyte migration and macrophage differentiation towards the inflammatory phenotype (Oh et al., 2018). Thus, PPB reduces monocyte-induced EC death and monocyte-induced VSMC proliferation and migration, and the effect of PPB on pyroptosis (leading to attenuation of these cells and aortic cell dysfunction) of endothelial cells and VSMCs in high-fat diet (HFD)-fed mice (Oh et al., 2020). In a high glyceride, high cholesterol environment, SIRT1 expression is suppressed, leading to reduced reverse cholesterol transport, mediating the differentiation of monocytes to foam cells and impeding the reduction of foam cells in atherosclerotic plaques (Kitada et al., 2016). Ox-LDL and saturated fatty acids are among the widely studied danger signals in atherosclerotic plaques.

**FIGURE 2:**
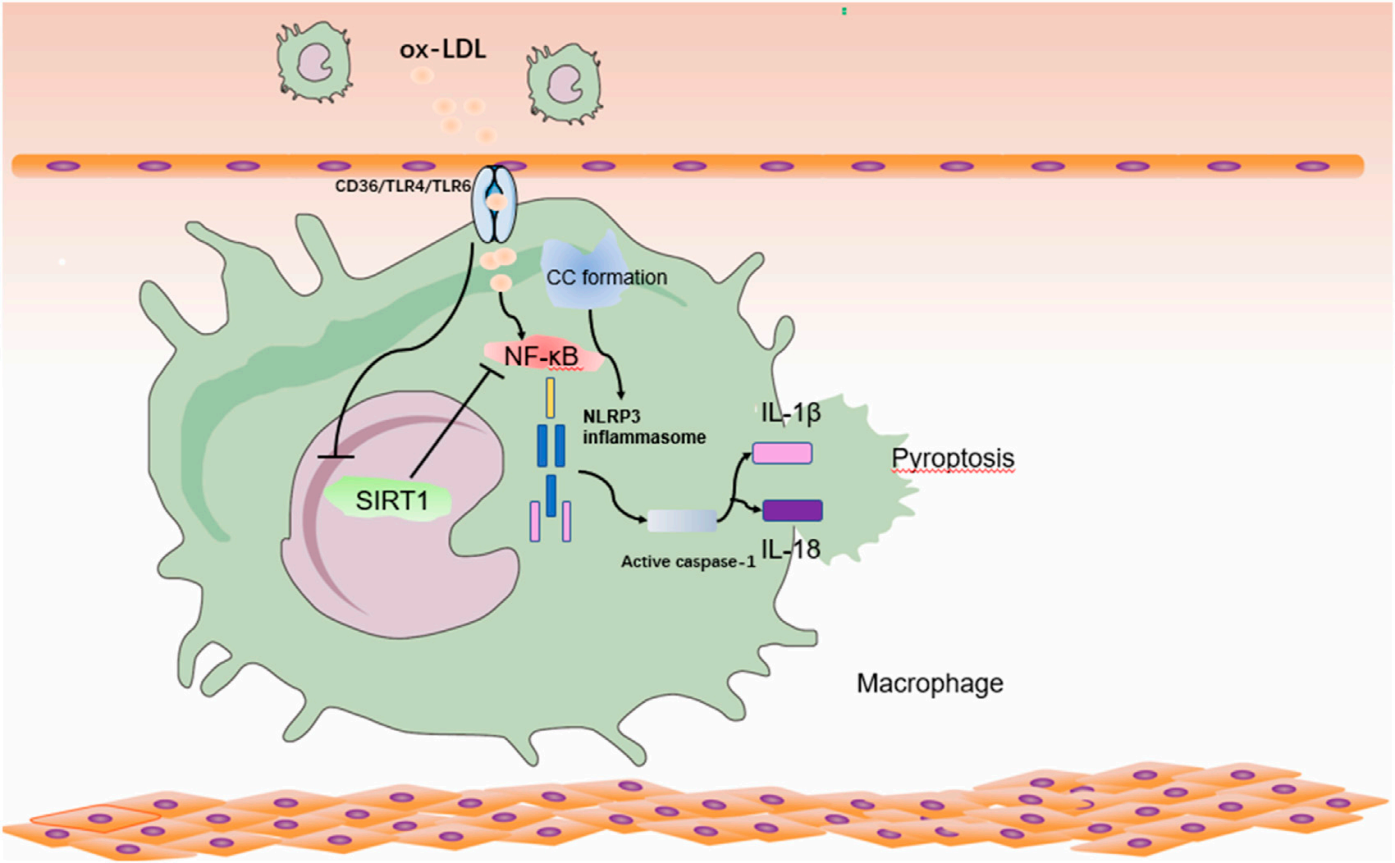
Relationship between SIRT1 and pyroptosis pathway in atherosclerosis in macrophages. Ox-LDL and CC- in atherosclerotic lesions activates NF-κβ in macrophages with NLRP3 inflammatory vesicle initiation and NLRP3 inflammatory vesicle activation, promoting the inflammatory factor IL-18/IL-1β, leading to cytoplasmic swelling and membrane rupture, resulting in the release of inflammatory factors and promoting the onset of pyroptosis. At the same time, ox-LDL inhibits the expression of SIRT1, which further leads to the activation of NF-κβ, and this vicious circle will further aggravate the development of AS. ox-LDL: oxidatively modified low-density lipoprotein; NF-κβ: nuclear factor kappa-light chain-enhancer of activated B.

Ox-LDL combines with macrophage CD36 to trigger TLR4/TLR6 assembly, leading to the formation of NF-κβ pathway (Stewart et al., 2010), promotes ROS release and initiates these cells for inflammatory vesicle NLRP3 activation. Ox-LDL can effectively initiate macrophages and thus activate cc-mediated NLRP3 inflammatory vesicles (Mason and Libby, 2015). Numerous studies have found that SIRT1 plays an important role in adipose tissue and inflammation. A study found that SIRT1 was proteolyzed in the adjacent tissue of congenitally obese mice, even after normal mice consumed high-fat foods. They also found that inflammation induced caspase-1 activation and subsequent shearing of SIRT1, thereby promoting metabolic dysfunction in adipose tissue (Chalkiadaki and Guarente, 2012). Activation of SIRT1 inhibits NF-κβ signaling pathway and reduces the development of atherosclerotic plaque inflammation and pyroptosis (Tabas and Bornfeldt, 2016) (Figure 2). Therefore, the study of SIRT1 inhibition of AS through the pyroptosis pathway has received much attention.

#### Sirtuins3

SIRT3 is a deacetylase that regulates the acetylation of most lysine in mitochondria (Sun et al., 2018). SIRT3 plays an important role in mitochondrial energy production and metabolic homeostasis (Dikalova et al., 2020). Disturbances in mitochondrial energy metabolism are responsible for AS and lead to abnormal energy expression and reduced ATP (Pircher et al., 2016; Dong et al., 2017). NAD^+^ plays a key role in ATP production in mitochondria and is an electron carrier (Matasic et al., 2018). Increased levels of NAD^+^ trigger the ability to express SIRT3 and lead to a regulatory pathway of target-specific deacetylation sirtuins and NAD^+^ play an important role in maintaining vascular homeostasis and preventing the development and progression of AS (Kane and Sinclair, 2018). SIRT3-mediated deacetylation modifies and activates long chain acyl-CoA dehydrogenase (LCAD), a key enzyme for fatty acid β-oxidation, and promotes fatty acid metabolism (Sun et al., 2018). Mitochondrial electron transfer chain (ETC) is a major source of ROS, and oxidative stress caused by excess reactive oxygen species production has emerged as an important and ultimate common mechanism of AS (Kattoor et al., 2017). Accelerated AS and elevated mitochondrial ROS were seen in ApoE^−/-^ mice deficient in the antioxidant system, suggesting a role for mitochondrial ROS in AS (Fang et al., 2021). SIRT3 enhances the ability of mitochondria to cope with ROS in multiple ways (Sun et al., 2018). NLRP3 inflammatory vesicles exacerbate AS formation, and its enhanced action exacerbates plaque instability (Hoseini et al., 2018). Since ROS production enhances the formation of NLRP3 inflammatory vesicles, inhibition of ROS can be used to protect against the pyroptosis of various diseases (Qiu et al., 2017). Increased SIRT3 activity blocks NLRP3 inflammatory vesicle activation and protects cells from oxidative stress (Cong et al., 2020).

#### Others

The Sirtuin family, from SIRT1 to SIRT7, plays an essential role in all phases of AS (Tao et al., 2019). SIRT2 has similar or opposite functions in AS and its pathogenesis. Two activators of SIRT2, resveratrol and NAD^+^, inhibit ASC approach to NLRP3 and thus inhibit NLRP3 inflammatory vesicle assembly (Zeng et al., 2019). Recent studies have identified an important role for SIRT2 in oxidation and antioxidation in vascular disease (Liu et al., 2019). Oxidation of lipids and proteins has been measured in various cardiovascular diseases and the extent of oxidation is strongly correlated with disease development, suggesting a role for oxidative stress in the development of AS (Stocker and Keaney, 2004; Förstermann et al., 2017). Sirtuin6 (SIRT6) is an evolutionarily conserved nicotinamide adenine dinucleotide-dependent histone deacetylase (Zi et al., 2019). SIRT6 acts mainly through histone-3 deacetylation in the promoter region of its target genes, including apoptosis, inflammation and lipid metabolism, which are key pathways regulated during AS (Grootaert et al., 2021). SIRT6-deficient (SIRT6^−/−)^ human MSCs exhibit increased levels of oxidative stress, reduced redox capacity, and increased sensitivity to oxidative responses (Pan et al., 2016). Meanwhile, SIRT7 plays a role in lipid metabolism and cardiomyopathy (Wang et al., 2021). The sirtuin family has been studied in neovascular diseases such as cardiomyopathy, endothelial dysfunction, and AS (Winnik et al., 2015). Studying the role of the sirtuin family in AS through pyroptosis remains a challenge.

### microRNA and Pyroptosis

#### microRNA and Endothelial Cells Pyroptosis

With the development of sequencing technology, researchers have gradually discovered that miRNA plays an important role in AS endothelial cells pyroptosis. Pyroptosis in the same cell model can be regulated by different miRNAs by acting on their corresponding single or multiple target genes. Recently, Chen et al. showed that miR-20a expression was downregulated in ox-LDL-induced human aortic endothelial cells (HAECs), so that its overexpression could target the negative regulation of TLR4 and thioredoxin-interacting protein (TXNIP), which in turn inhibited downstream NLRP3 activation and expression of ASC, IL-1β and other pyroptosis-related proteins to protect cells from ox-LDL-induced pyroptosis and inflammatory damage and reduce the risk of AS (Chen et al., 2018). Similarly, miR-30c-5p was reported to inhibit NLRP3-mediated cell pyroptosis with FOXO3 as a downstream target in ox-LDL-treated HAECs, but did not show effective modulation in NLRP3 activation dependent on TLR signaling (Li et al., 2018). It has also been shown that miRNA levels are positively correlated with the onset of pyroptosis (Yao et al., 2020). Zeng et al. found in ox-LDL-stimulated vascular endothelial cells that miR-125a-5p inhibited TET2 expression from the post-transcriptional level, leading to abnormal DNA methylation, abnormal mitochondrial function metabolism and increased ROS generation, activation of NF-κβ to induce activation and maturation of inflammatory vesicles and release of pro-inflammatory factors IL-1β and IL-18, and enhanced cell pyroptosis to promote AS (Zhaolin et al., 2019b).

#### microRNA and Macrophage Pyroptosis

As one of the important links in the development of AS, the pyroptosis of macrophages can promote the formation of a necrotic cellular core, leading to plaque rupture and increased instability in the late stage of AS and this process is also regulated by miRNAs. Different miRNAs can exert contradictory effects due to the various types of targets they bind. It was found that miR-223 inhibits NLRP3 translation by combining with a conserved binding sites within the 3′ non-coding region of NLRP3 mRNA (Bauernfeind et al., 2012). Wang et al. subsequently used ox-LDL and LPS to stimulate human THP-1-derived macrophages to establish an AS inflammation model and found that miR-9 could inhibit NLRP3 inflammasome activation and reduction of inflammatory response via JAK1/STAT1 signaling pathway further stabilize atherosclerotic plaque stability (Wang et al., 2017). Some studies have also demonstrated that miR-181a can engage with the MEK1 3ʹ non-coding region, inhibit its molecular machinery expression, and reduce ox-LDL-induced expression of NLRP3 and other inflammatory vesicles in THP-1-derived macrophages via the MEK/ERK/NF-κβ pathway, and then inhibits pyroptosis (Song et al., 2019).

In contrast, some miRNAs can induce macrophage pyroptosis to promote AS. One study found elevated miR-33 expression in atherosclerotic plaques that targeted multiple genes involved in regulating cholesterol efflux and fatty acid oxidation processes, including Prkaa1 (Price et al., 2019). Its gene product AMPK downregulates DNA glycosylase OGG1, which enhances mitochondrial DNA damage in macrophages and cellular scorching in atherosclerotic plaques, leading to accelerated AS progression (Tumurkhuu et al., 2016). Likewise, upregulation of miR-155 in the ApoE^−/-^ murine AS model exacerbated atherosclerotic lesions and promoted activation of NLRP3 inflammatory vesicles and inflammatory factors such as IL-18 and IL-1β. Meanwhile, regulation of ERK1/2 activity is associated with cardiovascular disease, and inhibitors decreased ERK1/2 activity and increased aortic elastin content, suggesting the feasibility of ERK1/2 inhibitors in the treatment of AS with reduced arterial elastin content (Zeng et al., 2021). *In vitro* experiments revealed that the molecular mechanism of miR-155 enhanced NLRP3 expression in macrophages through mediating the ERK1/2 pathway (Yin et al., 2019).

#### microRNA and Smooth Muscle Cells Pyroptosis

Smooth muscle cells pyroptosis in AS can contribute to plaque fibrous cap rupture damage to an unstable state, leading to acute coronary events. Recently, miRNAs have been reported to be involved in the regulation of these mechanisms. Zhong et al. found that inflammation caused aberrant methylation and low expression of the miR-145 promoter in VSMCs of the aortic wall through *in vitro* and *in vivo* studies; low levels of miR-145 attenuated the inhibition of the CD137/NFATc1 axis, further activating NLRP3 inflammatory vesicles to promotes the release of inflammatory mediators and accelerates the onset of AS (Zhong et al., 2018). Overexpression of reduced levels of miR-125a-5p in human vascular smooth muscle cells cultured with ox-LDL as an *in vitro* model of AS revealed that miR-125a-5p directly downregulates the target gene C-C motif chemokine 4-like (CCL4), which in turn reduces the expression of NLRP3, IL-1β and other proteins, confirming the key regulatory role of miR-125a-5p on pyroptosis-related proteins and inflammation during AS (Jiawang Wang et al., 2019).

In conclusion, it is the multispecies, multicellular, and multitarget mechanisms of miRNAs that allow them to exhibit opposite effects on pyroptosis in AS. Taking advantage of this property, continuing to explore the biological functions of miRNAs will surely provide a theoretical basis for revealing the underlying pathophysiological mechanisms of AS (Table 1).

**TABLE 1:** miRNA involved in the regulation of pyroptosis-related molecules.

miRNA	Model	Signaling pathways/Target	Result	References
miR-20a	Human aortic endothelial cells (HAECS)	TLR、TXNIP	Negatively regulates TLR4 and NLRP3 signaling to protect HAECs from inflammatory injuries, inhibits the atherosclerotic development	Chen et al. (2018)
miR-30c-5p	HAECs	FOXO3	Inhibits NLRP3 inflammasome-mediated endothelial cell pyroptosis, anti-atherosclerosis	Li et al. (2018)
miR-125a-5p	Human umbilical vein endothelial cells	TET2	Activates NLRP3 inflammasome and caspase-1, promotes pyroptosis and atherosclerosis progression	Zhaolin et al. (2019b)
	Human vascular smooth muscle cells	CCL4	Inhibits expression of NLRP3、ASC、caspase-1 and IL-1β proteins, anti-atherosclerosis	Jiawang Wang et al. (2019)
miR-223	Primary macrophages	NLRP3	Inhibits NLRP3 inflammasome	Bauernfeind et al. (2012)
miR-9	Human THP-1 derived macrophages	JAK1/STAT1	Inhibits activation of the NLRP3 inflammasome, attenuates atherosclerosis-related inflammation	Wang et al. (2017)
miR-181a	THP‐1 macrophages	MEK1	Inhibits the expression of NLRP3 inflammasome-related proteins, alleviates pyroptosis	Song et al. (2019)
miR-33	Bone marrow derived macrophages, Ldlr^**−/−**^ mice	Prkaa1	Promotes pyroptosis and atherosclerosis progression	Tumurkhuu et al. (2016)
miR-155	THP-1 macrophages, ApoE^−/-^ mice	ERK1/2	Increases the expression of NLRP3 inflammasome and the secretion of IL-1β and IL-18, aggravates the carotid atherosclerosis lesion	Yin et al. (2019)
miR-145	Vascular smooth muscle cells, ApoE^−/-^ mice	CD137/NFATc1	Inhibits NLRP3 inflammasome and pyroptosis, increases the stability of atherosclerosis lesions	Zhong et al. (2018)

TLR, toll-like receptors; TXNIP, thioredoxin-interacting protein; FOXO3, Forkhead box O3; TET, ten-eleven translocation; CCL4, C-C motif chemokine 4-like; JAK1, Janus kinase 1; MEK1, MAP, kinase 1; Prkaa1, AMP, kinase; NFATc1, nuclear factor of activated T cells1.

## Conclusions and Perspectives

Epidemiology shows that cardiovascular mortality and disability rates are increasing annually. Most cardiovascular diseases are determined by vascular diseases, such as AS and stroke. Cardiovascular diseases have become a serious threat to human health, and have become one of the key concerns in the treatment of diseases. In recent decades, numerous studies have identified the role of pyroptosis in AS. AS is a complex, multi-step process that affects blood flow through luminal narrowing or thrombosis, leading to numerous diseases such as myocardial infarction, stroke and various other cardiovascular diseases. A large reason for the development of vascular disease is related to impaired endothelial cell function, which is the initial initiating factor of AS. Endothelial cell injury and dysfunction lead to LDL deposition and oxidative reactions to secrete adhesion factors such as VCAM-1 and ICAM-1, which promote the interaction of monocytes with endothelial cells and their subsequent transfer to the endothelial layer, where monocytes mature into macrophages that further evolve into foam cells, promoting the formation, progression and impaired rupture of atherosclerotic platelets in AS.

Pyroptosis is an inflammatory response of cells, and the released inflammatory factors can cause the emission of more inflammatory cells, further enhancing the ability and intensity of the inflammatory response, thus creating a vicious cycle that exacerbates the development of AS. These studies are currently a promising strategic direction to inhibit the development of AS by affecting pyroptosis, such as the sirtuin family, NF-κβ. However, the underlying mechanisms of pyroptosis and AS, intercellular transformation, and molecular mechanisms are still unclear. Therefore, a more profound exploration of the potential mechanisms and regulatory targets of cell pyroptosis in AS would be an innovative strategy for AS treatment.
